# Hypoxia-Inducible Factor-1α Polymorphisms and Risk of Cancer Metastasis: A Meta-Analysis

**DOI:** 10.1371/journal.pone.0070961

**Published:** 2013-08-28

**Authors:** Qian Zhang, Yan Chen, Bin Zhang, Bin Shi, Wenjun Weng, Zhipeng Chen, Nannan Guo, Yibing Hua, Lingjun Zhu

**Affiliations:** 1 Department of Oncology, The First Affiliated Hospital of Nanjing Medical University, Nanjing, China; 2 Department of Emergency, The First Affiliated Hospital of Nanjing Medical University, Nanjing, China; 3 Department of Thoracic and Cardiac Surgery, The First Affiliated Hospital of Nanjing Medical University, Nanjing, China; 4 Department of Gastrointestinal Surgery, The First Affiliated Hospital of Nanjing Medical University, Nanjing, China; Sapporo Medical University, Japan

## Abstract

**Background:**

HIF-1α is a major regulator in tumor progression and metastasis which responds to hypoxia. Many studies have demonstrated that hypoxia-inducible factor1-α (HIF-1α) polymorphisms are significantly associated with cancer metastasis, but the results are inconsistent. We conducted a comprehensive meta-analysis to estimate the associations between HIF-1α C1772 T polymorphism and cancer metastasis.

**Methods:**

Comprehensive searches were conducted on PubMed and EMBASE database. Fifteen studies were included in the meta-analysis. We used the OR and 95%CI to assess the associations between HIF-1α C1772T polymorphism and cancer metastasis. Heterogeneity and publication bias were also assessed by Q test, *I* ^2^, and funnel plot.

**Results:**

Totally, fifteen studies including 1239 cases with metastasis-positive (M+) and 2711 cases with metastasis-negative (M−) were performed in this meta-analysis. The results showed that HIF-1a C1772T polymorphism was associated with the increased risk of cancer metastasis (T allele vs. C allele, OR  = 1.36, 95% CI  = 1.12–1.64; TT+ TC vs. CC, OR  = 1.39, 95% CI  = 1.13–1.71; TT vs. TC+ CC, OR  = 1.93, 95% CI  = 0.86–4.36). In the subgroup analyses, the significant associations remained significant among Asians, Caucasians and other cancers in the dominant model. Publication bias was not observed in the analysis.

**Conclusions:**

Our results indicate that the HIF-1αC1772T polymorphism T allele may increase the risk of cancer metastasis, which might be a potential risk factor of cancer progress.

## Introduction

Cancer metastasis is a progress that tumor cells displace from the primary site to distance site where cancer cells adapt to a tissue microenvironment and is the most important cause of death in cancer patients [1]. The development of metastasis consists of a series of complex steps which involve in immunologic escape, angiogenesis, invasion of lymph- and blood vessels and so on [2]. The molecular mechanisms of angiogenesis on cancer metastasis have become focal points in the past years. Recently, research of tumor metastasis has been focused on the hypoxic condition. Hypoxia is one of the most important mechanisms that induces cancer metastasis [3]–[6] and regulates the metastatic process by metabolism, angiogenesis, innate immunity, and stem cells induction [4]. Hypoxia-inducible factor1α (HIF-1α) plays an important role in the growth and metastasis of tumor. Previous study has showed that increased level of HIF-1α protein was associated with lymph node metastasis and high malignant degree [7].

Genetic polymorphisms have been considered as the main genetic elements involved in the occurrence and development of cancer [8]. HIF-1α C1772T (rs11549465) is a common single nucleotide polymorphism (SNP) that is located in exon 12 which results in proline to serine. The polymorphism promotes the development and progression of cancer by increasing the density of cancer microvessel [9]. A number of studies have showed the relationship between the HIF-1α C1772T polymorphism and cancer metastasis, but the results have been disparate [29], [10]–[23]. No meta-analysis has been performed to investigate these associations so far. To better explore the associations of HIF-1α C1772T polymorphism with cancer metastasis, we conducted a meta-analysis to collect and analyze the published data.

## Materials and Methods

### Literature source and search strategy

The published case-control studies that investigated the associations between the HIF-1α C1772T polymorphism and cancer metastasis were searched on PubMed and EMBASE database (between January 1, 2005 and December 1, 2012). The keywords and terms used for this search were “HIF-1 OR hypoxia-inducible factor-1”, “polymorphism” AND “cancer”. In addition, references of retrieved publications were also screened by hand-searched. Studies included in the current meta-analysis have to meet the following inclusion criteria: (i) independent case-control design, (ii) evaluation of the associations between HIF-1α C1772T polymorphism and cancer metastasis, and (iii) provide available genotype frequency.

### Data Extraction

Two investigators independently extracted data. If the data was different, the third reviewer was asked to check until the data was right. For each eligible study, we collected the following information: first author's name, the year of publication, country of origin, cancer type, ethnicity of study population, the number of metastasis-positive (M+) and metastasis-negative (M−) cases and genotypes. The criteria of metastasis-positive (M+) and metastasis-negative (M−) depended on TNM [24]. The patients were assigned to metastasis-positive (M+) depended on the presence of detectable lymph nodes metastasis or distant metastasis at the time of diagnose or follow-up, or assigned to metastasis- negative (M−). In one study, if lymph nodes metastasis and distant metastasis were both investigated, we selected lymph nodes metastasis as the criteria in the current meta-analysis.

### Statistical analysis

The strength of the association between HIF-1α C1772 T and cancer metastasis was measured by odds ratio (OR) and 95% confident interval (CI). The influence of study size of evaluated studies on the results was assessed by the Weight. The statistical significance of the pooled OR was determined using the Z-test (*P* <0.05 was considered significant). The pooled OR was first evaluated on allele (T allele vs. C allele), and then on the dominant model (TT+CT vs. CC) and recessive model (TT vs. CT+CC). Subgroup analysis were also conducted by ethnicity, cancer types (if one cancer type contained one individual study, it was combined into the “other cancers” group).

Heterogeneity between studies was assessed by the χ^2^-based Q-test and *I* ^2^. If *P*>0.05 of the Q -test indicated a lack of heterogeneity across eligible studies, a fixed-effects model (the Mantel-Haenszel method) was used for meta-analysis. Otherwise, the random-effects model was used (DerSimonian and Laird method). Funnel plot and Egger's linear regression test were applied to evaluate the potential publication bias. All statistical analyses were carried out with Stata software (version 11.0, USA), using two-sided *P*-values.

## Results

### Characteristics of studies

**Figure 1:**
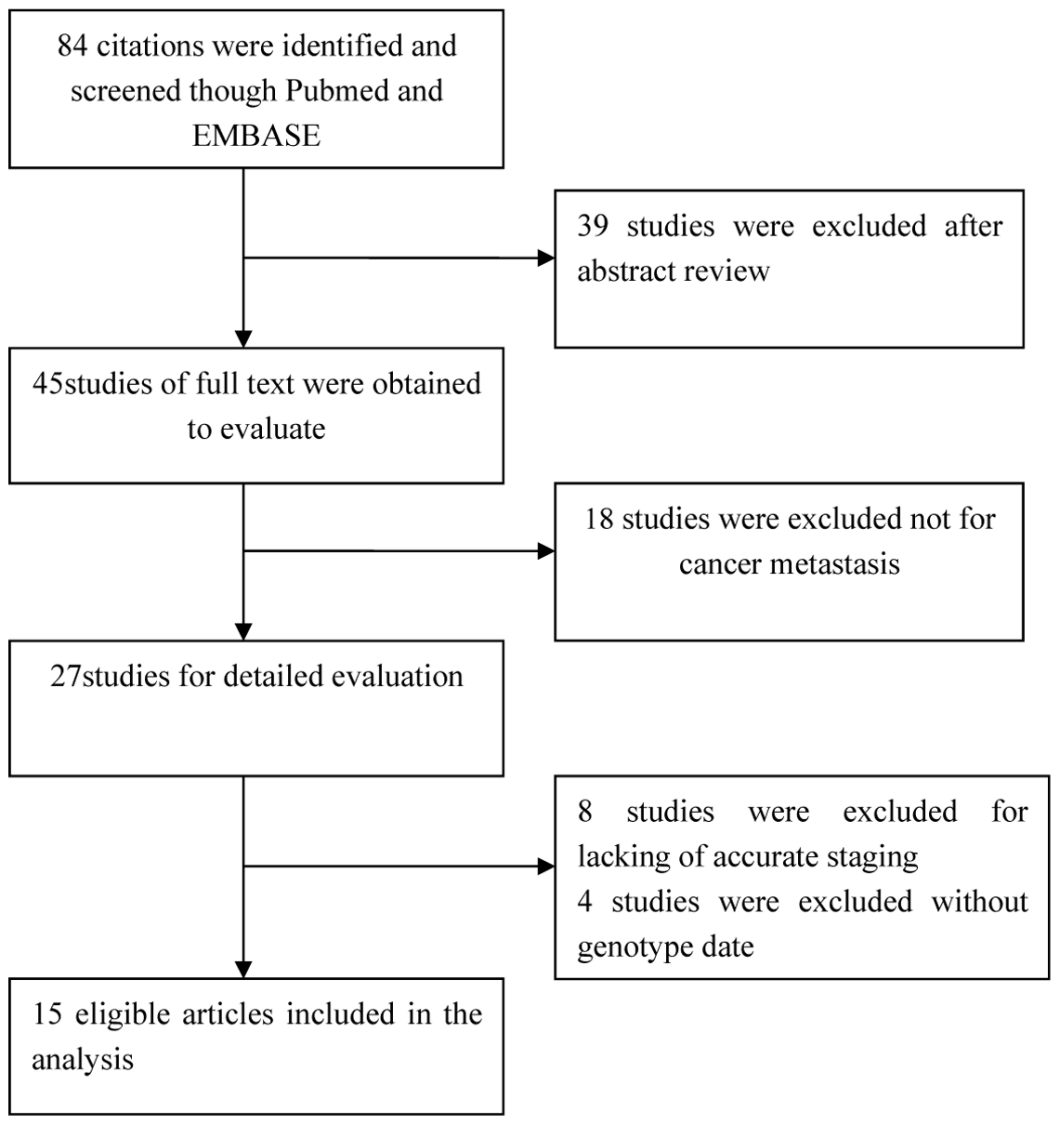
The detailed process of identifying eligible studies.

27 studies were obtained to evaluate the relationship between HIF-1α C1772 T and cancer metastasis. Twelve of them were excluded (8 studies were excluded for lacking of accurate staging, 4 studies without genotype data). Finally, 15 articles [29], [10]–[23] were included in our meta-analysis (Figure 1). The detail characteristics of eligible studies were summarized in Table 1. Among these studies, there were 10 studies of Asians and 5 of Caucasians. All studies were case-control studies which contained four studies with breast cancer, two with colorectal cancer and other cancers group. As shown in Table 1, 1239 (M+) cases and 2711 (M−) cases were included in the study.

**Table 1:** Characteristics of HIF-1a polymorphisms Included in the Meta-analysis.

Study	Year	Cancer type	Country	Ethnicity	Total		Genotypes					
					M(+)	M(−)	M(+)			M(−)		
							CC	CT	TT	CC	CT	TT
Apaydin	2008	Breast	Turkey	Caucasian	75	27	58	17	-	21	6	-
Kim	2008	Breast	Korea	Asian	48	42	45	3	-	36	6	-
Lee	2008	Breast	Korea	Asian	336	642	298	37	1	583	54	5
Naidu	2009	Breast	Malaysia	Asian	187	215	127	60	-	166	49	-
Knechtel	2010	Colorectal	Austria	Caucasian	221	127	174	47	-	106	21	-
Kang	2011	Colorectal	Korea	Asian	24	26	19	5	-	19	7	-
Ling	2005	ESCC^a^	China	Asian	25	24	16	9	-	23	1	-
Orr-Urtreger	2007	Prostate	Israel	Caucasian	3	371	2	1	0	262	96	13
Hsiao	2010	Hepatocellular	China	Asian	5	97	5	0	-	89	8	-
Shieh	2010	OSCC^b^	China	Asian	106	199	99	7	-	183	16	-
Wang	2011	Pancreatic	China	Asian	127	136	98	29	-	111	25	-
Qin	2012	Renal cell	China	Asian	26	594	25	1	-	547	47	-
Fraga	2011	UADTC^c^	Brazil	Caucasian	26	26	6	18	2	15	11	0
Mera-Menendez	2012	Larynx	Spain	Caucasian	12	106	6	1	5	79	17	10
Chai	2010	Cervical	China	Asian	18	79	10	7	1	55	18	6

a ESCC esophageal squamous cell carcinoma.

b OSCC Oral squamous cell carcinoma.

c UADTC upper aerodigestive tract cancer.

### Quantitative data synthesis

**Figure 2:**
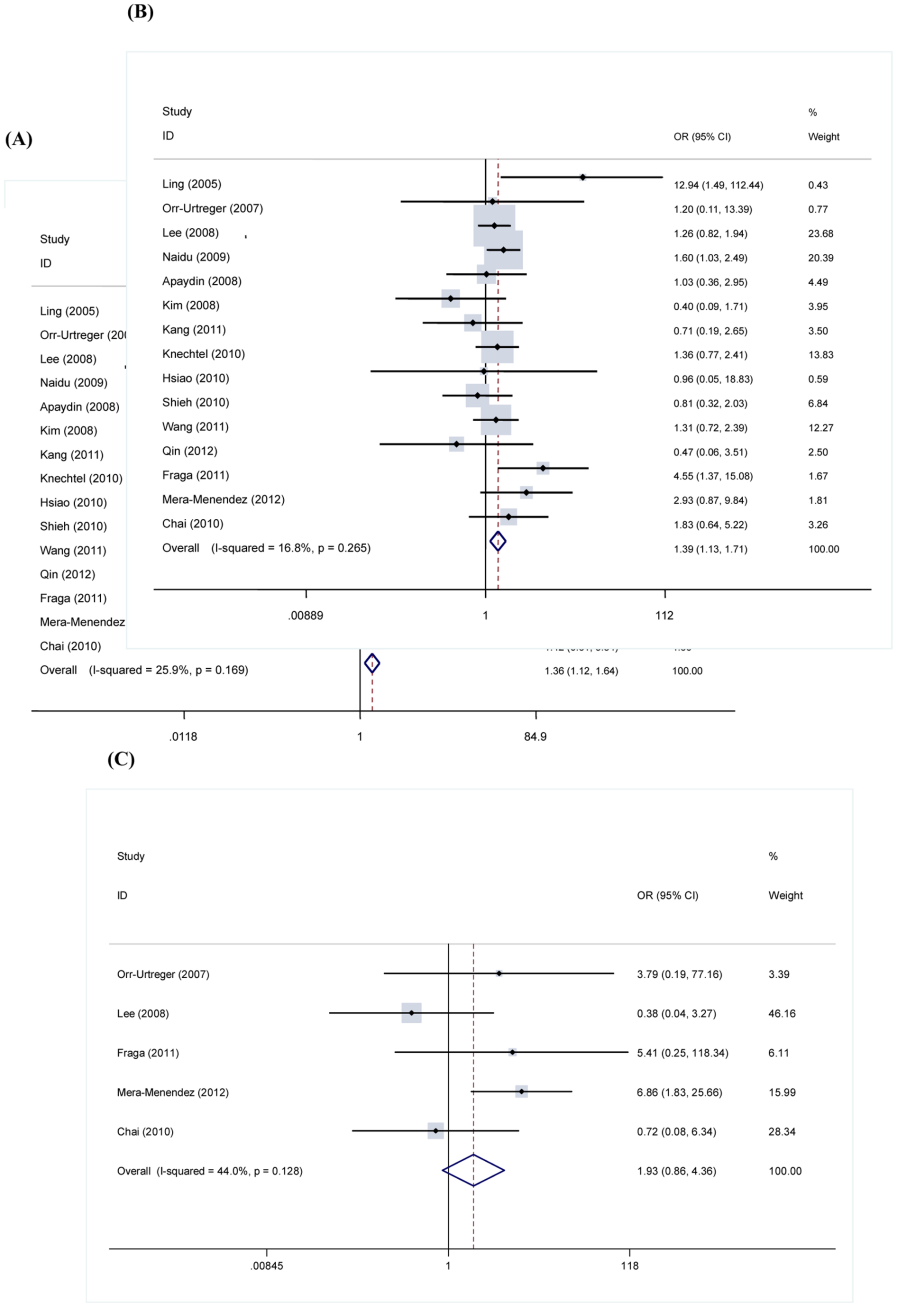
Forest plot of cancer metastasis associated with the HIF-1a C1772 T polymorphisms. (A) C allele vs. T allele. (B) The dominant model. (C) The recessive model. A fixed-effects model was used. The squares and horizontal lines correspond to the study-specific OR and 95% CI. The area of the squares reflects the weight (inverse of the variance). The diamond represents the summary OR and 95% CI.

We assessed the associations between the HIF-1α C1772T polymorphism and cancer metastasis. Overall, when all the eligible studies were pooled into the meta-analysis, variant T allele significantly increased the risk of cancer metastasis, compared with the wild-type C allele (OR  = 1.36, 95% CI  = 1.12–1.64, *P* = 0.002, *P*_heterogeneity_ = 0.17. *I* ^2^ = 25.9; Figure 2. A), the dominant mode (TT+CT vs. CC) showed that there were significant associations between HIF-1α C1772 T and cancer metastasis (OR  = 1.39, 95% CI  = 1.13–1.71, *P* = 0.002, *P*_heterogeneity_ = 0.27. *I* ^2^ = 16.8%; Figure 2. B). Significant associations were not observed under the recessive model (TT vs. CC+CT) (OR  = 1.93, 95% CI  = 0.86–4.36, *P* = 0.11, *P*_heterogeneity_ = 0.13. *I* ^2^ = 44%; Figure 2. C). The pooled OR for C vs. T and TT/CT vs. CC indicated that HIF-1a C1772T was significantly associated with a increased risk of cancer metastasis.

Under the dominant model, subgroup analysis based on cancer type was performed, significant associations were not found in colorectal cancer and breast cancer, but a significant association in other cancer was observed. In the stratified analysis by ethnicity, significant associations were observed among Asians and in Caucasians (Table 2).

**Table 2:** Stratified analysis of HIF-1a polymorphisms on cancer metastasis.

	N	M(+)	M(−)	OR(95%CI)	*P*	*P _heterogeneity_*	*I* ^2^(%)
Total	15	1239	2711	1.39 (1.13–1.71)	0.002	0.27	16.8
Tumor site							
Breast**cancer	4	646	926	1.31 (0.98–1.75)	0.07	0.31	16.3
Colorectal**cancer	2	245	153	1.23 (0.74–2.07)	0.43	0.38	0
Other**cancer	9	348	1632	1.62 (1.13–2.31)	0.008	0.17	31.3
Ethnicity							
Asian	10	902	2054	1.31 (1.03–1.66)	0.025	0.28	18.2
Caucasian	5	337	657	1.65 (1.08–2.52)	0.02	0.3	18

### Tests for heterogeneity and Sensitivity

Totally, no significant heterogeneity was observed among studies for the associations between HIF-1α C1772 T polymorphism and cancer metastasis in the pooled analysis and stratified analysis of dominant model. The fixed-effects model was performed in the meta-analysis. Any single study was not found to change the pooled OR qualitatively by sensitivity analysis indicated that this meta-analysis is stable.

### Publication bias

**Figure 3:**
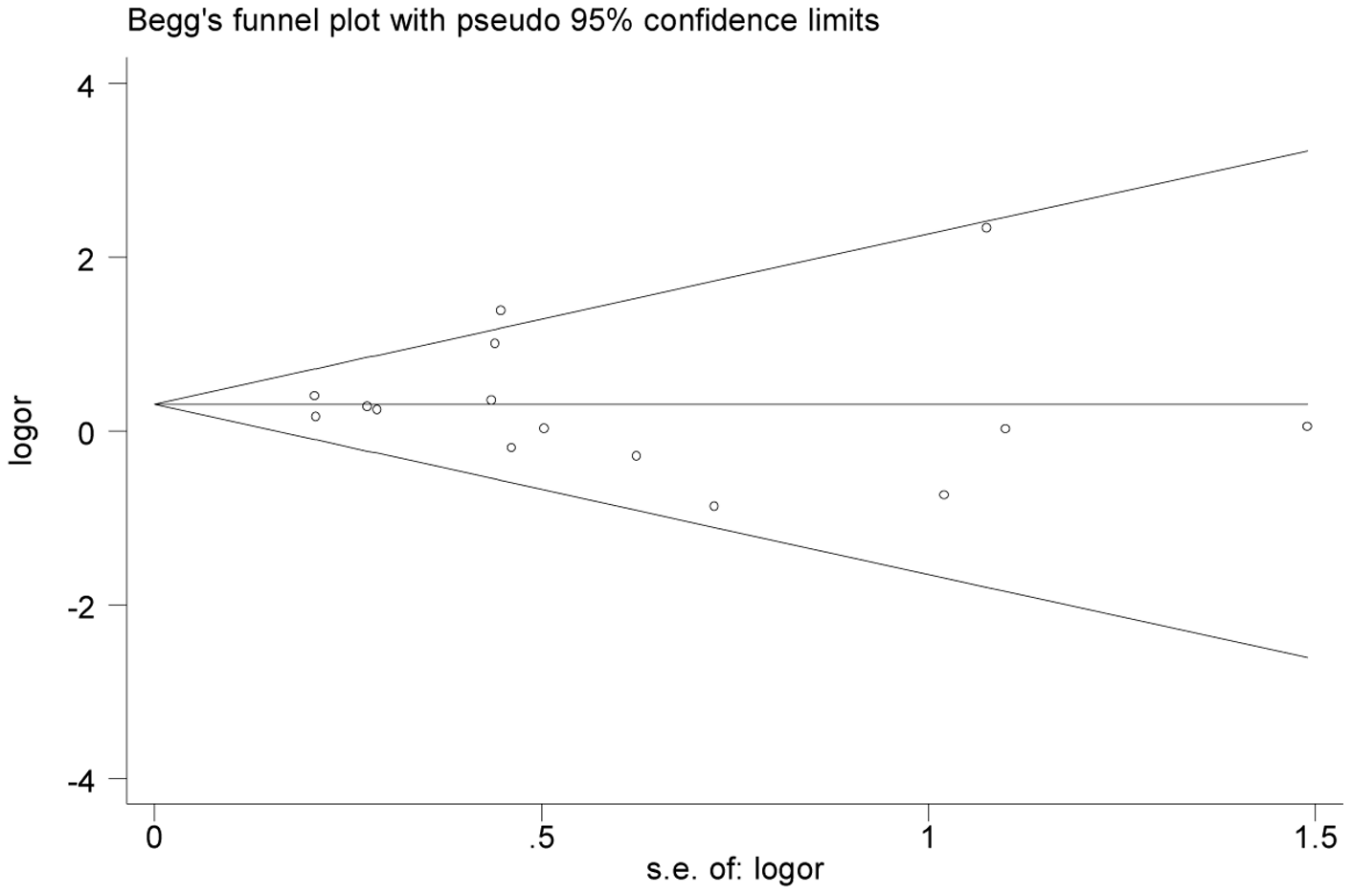
Begg's funnel plot for publication bias test(C allele vs. T allele). Each point represents a separate study for the indicated association.

Begg's funnel plot and Egger's test were performed to assess the publication bias of literatures. As shown in Figure 3, the shape of the funnel plot did not reveal any evidence of obvious asymmetry in all comparison models. Also, the results of Egger's test did not show any evidence of publication bias. (T vs. C, t = −0.13, *P* = 0.900, 95%CI  = −1.43–1.27; TT/TC vs. CC, t = 0.02, *P* = 0.983, 95%CI  = −1.23–1.26; TT vs. TC/CC, t = −0.73, *P* = 0.519, 95%CI  = −7.60–4.77).

## Discussion

The meta-analysis investigates the associations between HIF-1α C1772 T polymorphism and cancer metastasis. We found that variant T allele significantly increased the risk of cancer metastasis.

Hypoxia-inducible factor1α (HIF-1α) is a transcription factor was first found as a regulator of renal production of erythropoietin (Epo) [25]. It is a helix-loop-helix transcription factor that consists of α and β subunits. HIF-1α subunit is regulated by oxygen pressure and HIF-1 β is constitutively expressed [6]. In normoxic conditions, HIF-1α is hydroxylated at specific proline residues. Under hypoxic conditions, HIF-1α is induced and combines with the β subunit, then removes to the nucleus and initiates gene transcription [26], [27]. The HIF-1α protein contains five functional domains: basic helix-loop-helix (bHLH), Per/Arnt/Sim (PAS), N-terminal (N-TAD), C-terminal (C-TAD), and oxygen-dependent degradation (ODD) [28].

HIF-1α is overexpressed in regional or distant metastases and is also expressed higher in preneoplastic and premalignant lesions, indicating that overexpression of HIF-1α can occur very early in carcinogenesis which may become a potential biomarker of predicting tumor progress [29] and a good target for the detecting of tumor metastasis. HIF-1 activates the transcription of a large number of genes that for erythropoietin, vascular endothelial growth factor, endothelin-1, nitric oxide synthase, heme oxygenase-1, and insulin-like growth factor-2. However, the expression of vascular endothelial growth factor (VEGF) is an essential molecular event for tumor development and metastasis [30]. It is known that HIF-1 induces erythropoiesis and angiogenesis, and is also involved in the regulation of both vascular tone and glucose metabolism [31]. And HIF-1 transcriptional activity is regulated by the HIF-1α subunit. There were evidences indicate that HIF-1 plays an important role in cancer progression and metastasis [25], [30]. Higher expression levels of HIF-1α have been reported in human malignancies including colon, breast. HIF-1α polymorphism C1772T in human were initially identified in renal cell carcinoma patients which cause amino acid substitutions within the N-TAD, however, the difference in genotype distribution was not indicated between renal cell carcinoma cases and controls [32]. It has been reported that the C1772T polymorphism T allele in HIF-1α represent higher transcriptional activity than that of wild-type C allele under both normoxic and hypoxic conditions [33]. Therefore, the presence of this polymorphism might be associated with in cancer risk and cancer metastasis. However, the studies had controversial conclusions. A meta-analysis has proved that the HIF-1α C1772 T polymorphism is significantly associated with higher cancer risk [34]. The aim of this study was to investigate the association between HIF-1α C1772 T polymorphism and cancer metastasis. In our analysis, we found that variant T allele significantly increased the risk of metastasis. Further, the associations were very stable, which did not change apparently when the sensitivity analyses were performed. The results indicated that T allele is a potential risk factor in cancer metastasis. There was a significant association between HIF-1α C1772 T polymorphism and cancer metastasis under the dominant model, while no association was found in the recessive model. One possible explanation is that the number of studies which investigate the TT genotype separately is too small. The results may also indicate that heterozygous T has a stronger effect on an individual's phenotype than homozygous T. It is mean that individuals with CT genotype may have a higher risk of metastasis than those with TT genotype. Further larger sample size and well-designed studies should be performed to testimony our results.

For the stratification analyses based on ethnicity and cancer types. There was an evidence to indicate that the HIF-1α C1772T polymorphism was significantly associated with increased risk of cancer metastasis among Asians and Caucasians only for dominant genetic model. However, no significant association was found between the C1772T polymorphism and cancer metastasis in colorectal cancer and breast cancer under dominant model. Further studies using larger sample size are needed to validate. Kuwai et al. [35] indicated that the C1772T polymorphism in HIF-1α is not association with the progression and metastasis of colorectal cancer, while the study of patients with ESCC has a contrary result [16]. It may because C1772T has different values in different kinds of tumors. The T/T genotype is rare in our study, thus, the absence of association between T/T and cancer metastasis may be due to chance. Extended epidemiological studies would be needed to determine if this genotype is associated with cancer metastasis. There is a previous founding reported that HIF-1α genetic variant increases risk of breast Lymph node metastasis [18], which is consistent with our subgroup analysis. It is likely that variations of sample sizes may account for contradictory results.

There are some limitations and potential bias that must be acknowledged in our meta-analysis. First, only published studies were included in our studies, many unpublished data have been ignored in the analysis. Therefore, potentially publication bias will be existed in our results, although the statistical data did not reflect it. Second, because of lacking of detailed analysis about age, gender, smoking, drinking and so on, those potential factors may influence our results. Third, the number of the included studies was not large enough. So the statistical power is weak to evaluate the association between HIF-1a polymorphisms and metastasis, especially in stratified analyses.

In summary, our meta-analysis reveal that the HIF-1α C1772T polymorphism can increase the risk of cancer metastasis. Although some results of the analysis are limited by the small number of studies. Our results suggest that the polymorphism is a potential risk factor. Large sample size and well-designed studies are needed to evaluate our finding.
