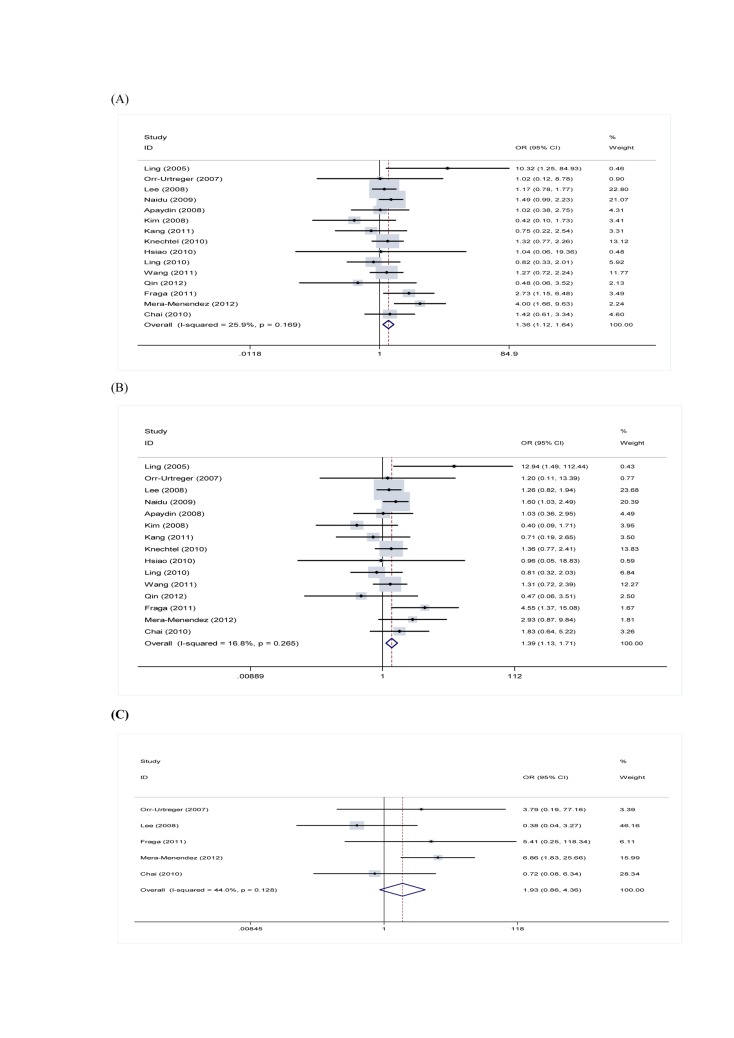# Correction: Hypoxia-Inducible Factor-1α Polymorphisms and Risk of Cancer Metastasis: A Meta-Analysis

**DOI:** 10.1371/annotation/938850a0-b05c-4557-becc-9d91d3d90742

**Published:** 2013-10-07

**Authors:** Qian Zhang, Yan Chen, Bin Zhang, Bin Shi, Wenjun Weng, Zhipeng Chen, Nannan Guo, Yibing Hua, Lingjun Zhu

The correct version of Figure 2 is available here: 

**Figure:**